# Potential molecular targets for intervention in pelvic organ prolapse

**DOI:** 10.3389/fmed.2023.1158907

**Published:** 2023-09-05

**Authors:** Xia Wu, Xiaochun Liu, Tingting Li

**Affiliations:** Third Hospital of Shanxi Medical University, Shanxi Bethune Hospital, Shanxi Academy of Medical Sciences, Tongji Shanxi Hospital, Taiyuan, China

**Keywords:** potential biomarkers, intervention, pelvic organ prolapse, micro-mechanism, molecular targets

## Abstract

Pelvic organ prolapse (POP) is a concerning gynecological benign illness in middle-aged and senior women. Its etiology is complex, the incidence rate is high, symptoms are clinically subjective, and its influence tends to be polarized. At present, for those who need medical treatment, whether surgical or non-surgical, complications cannot be ignored, and treatment effect needs to be optimized. However, there is a lack of accurate molecular biological interventions for the prevention, diagnosis, progression delay, and treatment of POP. Here, we reviewed the current state of understanding of the molecular mechanisms and factors associated with POP etiology. These factors include cyclins, matrix metal peptidases/tissue inhibitors of metalloproteinases, microRNAs, homeobox A11, transforming growth factor β1, insulin-like growth factor 1, fibulin 5, lysyl oxidase-like 1, oxidative stress, inflammatory response, estrogen, and other potential biomarkers associated with POP. In addition, relevant molecular targets that may be used to intervene in POP are summarized. The aim of this review was to provide more information to identify accurate potential biomarkers and/or molecular targets for the prevention, diagnosis, progression delay, and treatment of POP, with the goal of improving medical treatment for patients at-risk for POP or having POP. Continued research is needed to identify additional details of currently accepted molecular mechanisms and to identify additional mechanisms that contribute to POP.

## 1. Introduction

**Figure 1:**
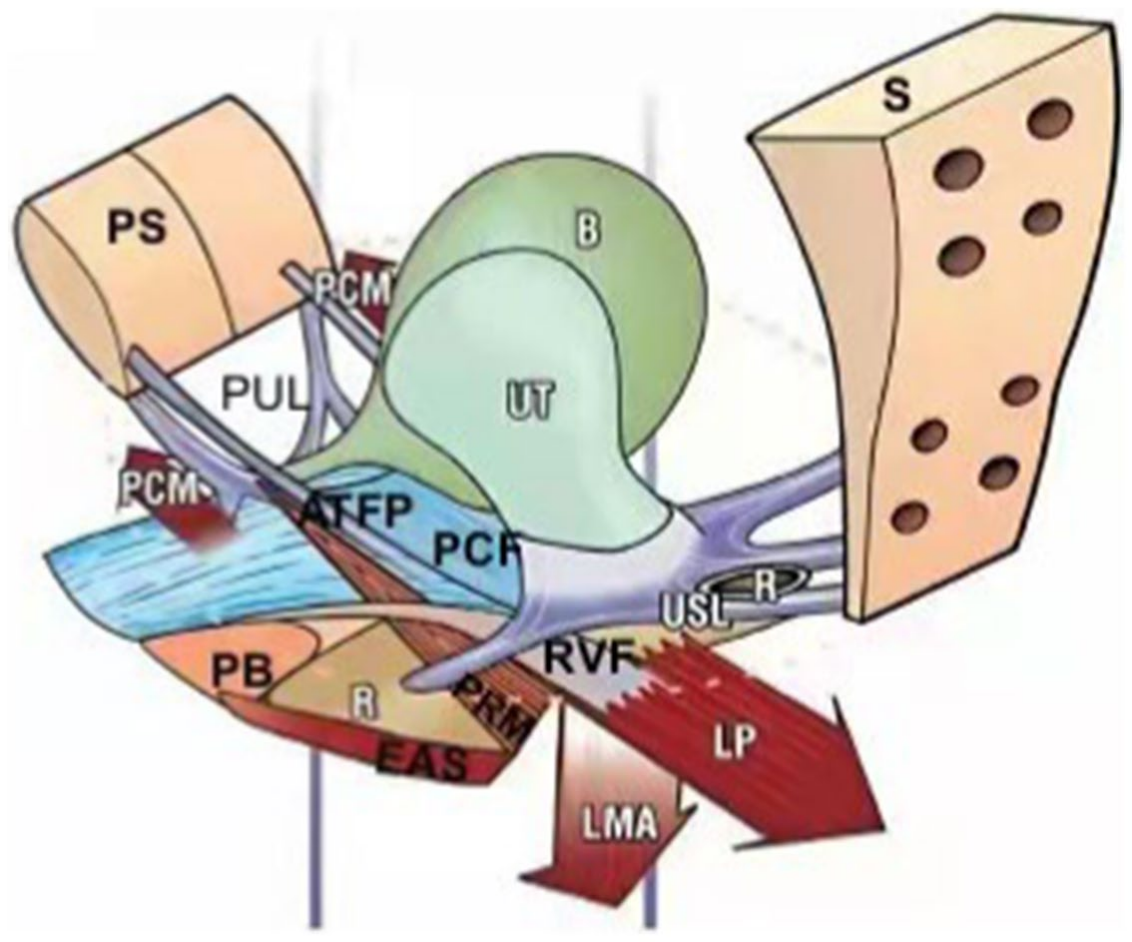
In 1990, Petros and Ulmsten first proposed the integral theory of the pelvic floor. It points out that different levels of vaginal support axis in different compartments together constitute an anatomical and functional organic whole, and weakening any structure will lead to the imbalance of the whole function, resulting in pelvic floor dysfunction disease. PS, pubic symphysis; PUL, pubourethral ligament; PCM, pubococcygeus muscle; ATFP, arcus tendineus fascia pelvis; PB, perineal body; PCF, pubocervocal fascia; EAS, external anal sphincter; PRM, pubic rectum muscle; RVF, rectovaginal fascia; LMA, longitudinal muscle of anus; LP, levator plate; USL, uterosacral ligament; S, sacrum; R: rectum; B, bladder; UT, uterus.

**Figure 2:**
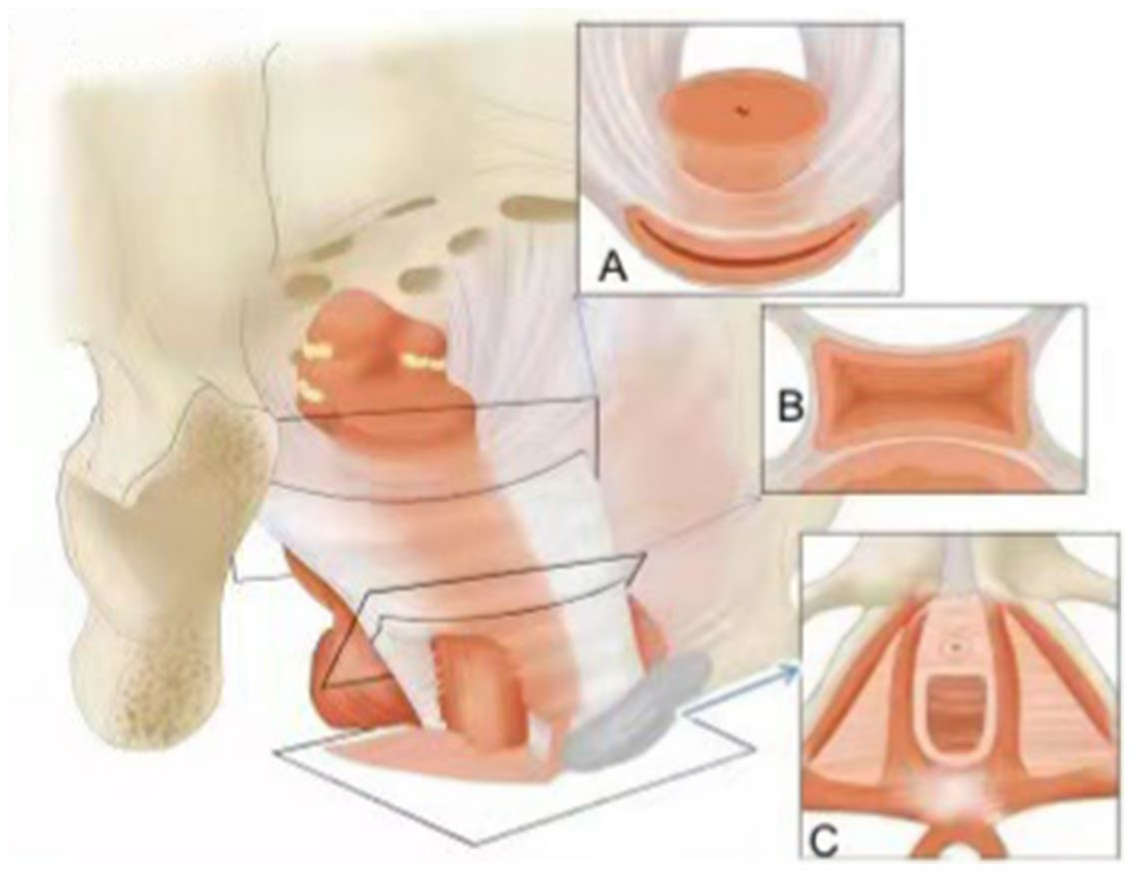
In 1994, theory of “Levels of Support” was proposed by Delancey, level 1 **(A)** is the upper supporting structure (main ligament-uterine low ligament complex); level 2 **(B)** is the lateral supporting structure (levator ani muscle group and bladder, rectovaginal fascia), level 3 **(C)** is the distal supporting structure (perineum and sphincter).

**Figure 3:**
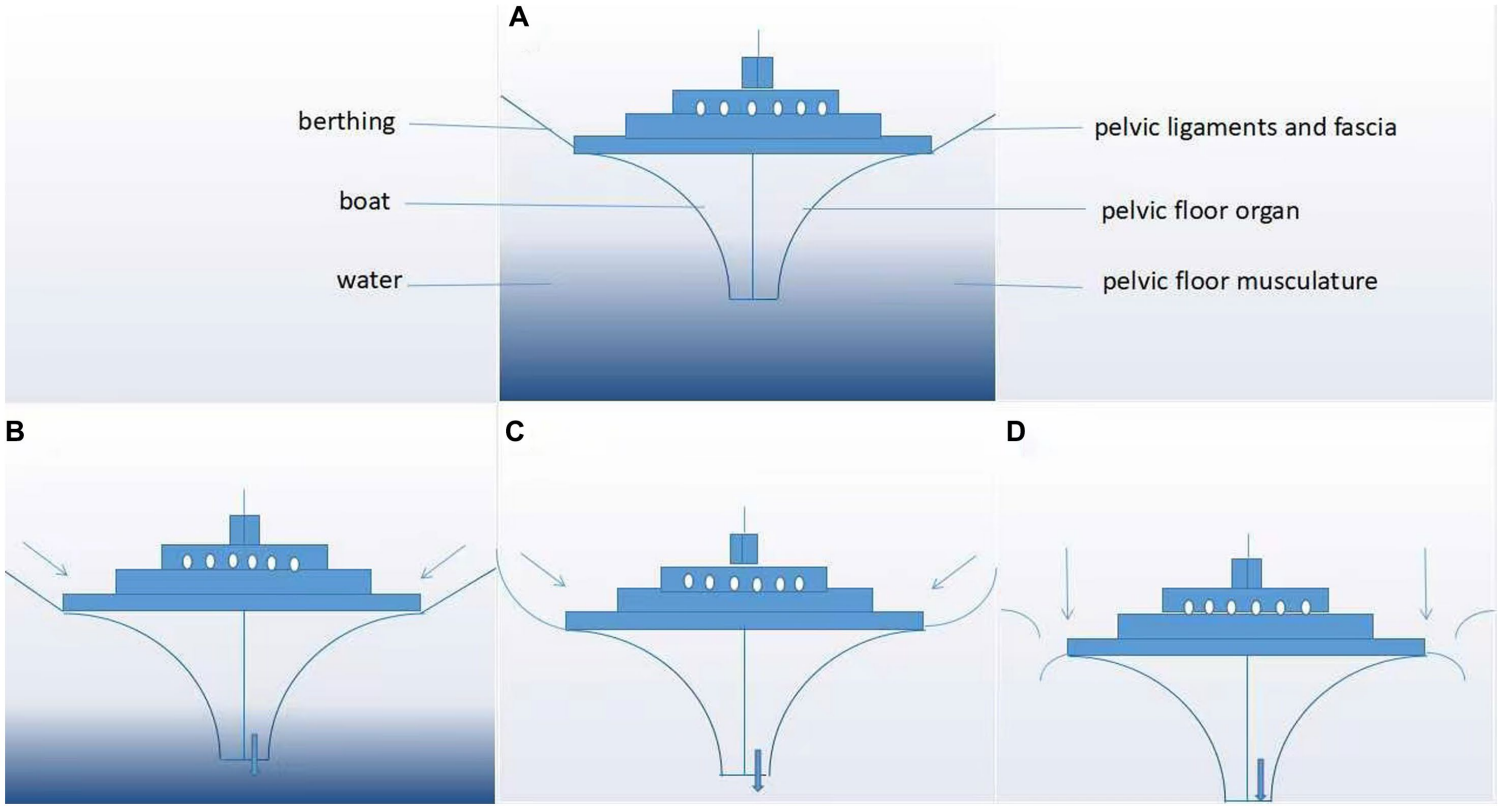
“Boat in dry dock” conception of pelvic floor disorders. As shown in panels **(A–D)**, the boat represents the pelvic floor organ, its berthing represents the pelvic ligaments and fascia, and the water represents the pelvic floor musculature. **(A)** Normal pelvic floor tissues and **(B–D)** evolution of pelvic organs after progressive damage to the pelvic floor support tissue.

**Figure 4:**
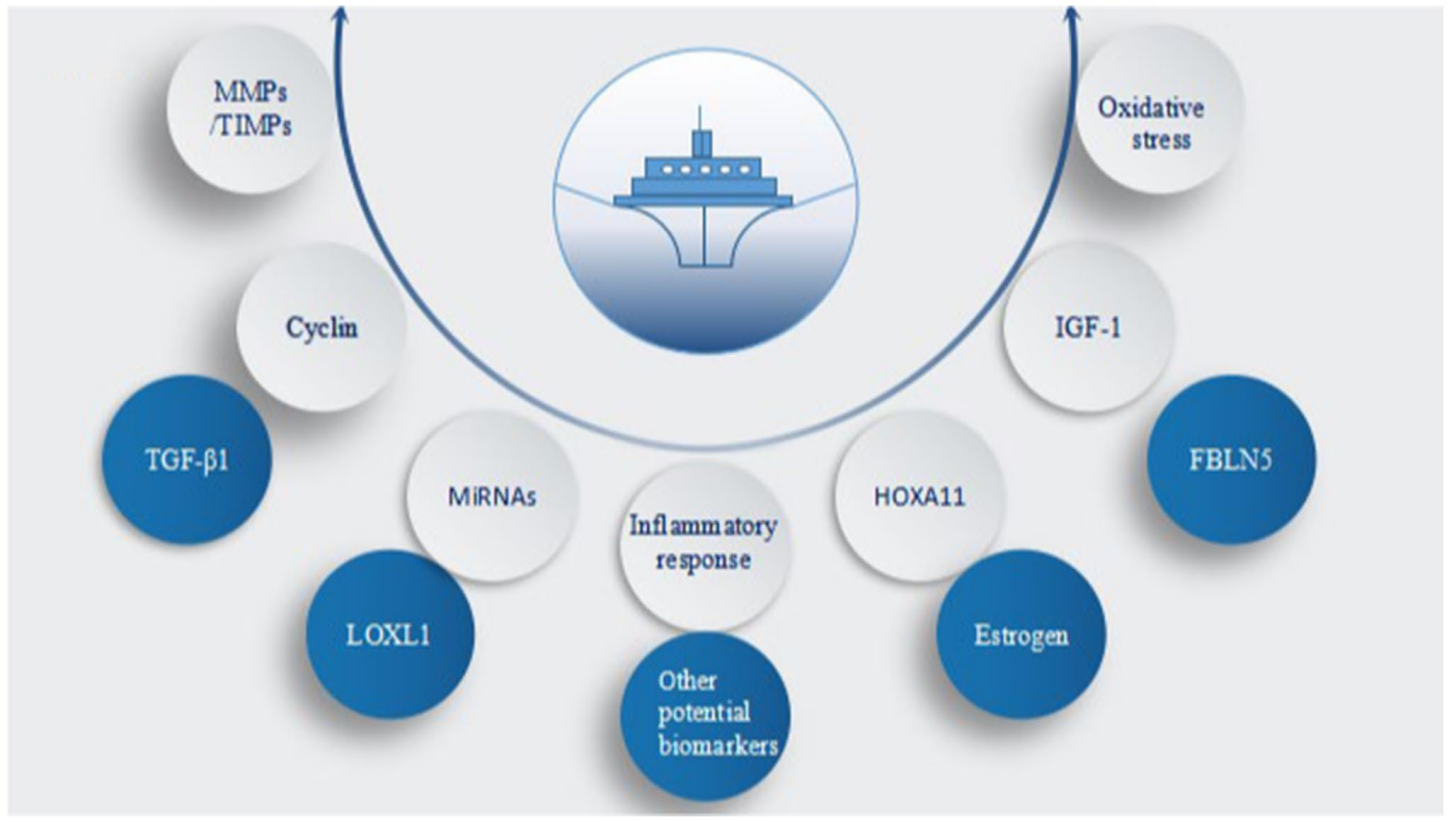
Core factors involved in pelvic organ prolapse (POP). MMPs/TIMPs, matrix metal peptidases/tissue inhibitors of metalloproteinases; TGF-β1, transforming growth factor β1; miRNAs, microRNAs; LOXL1, lysyl oxidase-like 1; HOXA11, homeobox A11; IGF-1, insulin-like growth factor 1; FBLN5, fibulin-5; oxidative stress, inflammatory response, estrogen, and other potential biomarkers.

The current understanding of the pelvic organ prolapse is mostly based on the integral theory of the pelvic floor proposed by Petros and Ulmsten, the theory of “Levels of Support” proposed by Delancey, and the theory of “Boat in dry dock” proposed by Norton (1–3) (Figures 1–3), Pelvic organ prolapse (POP) is considered to be a disease of pelvic floor defects caused by vulnerability of the support structure owing to diverse factors, and then leads to the decline and displacement of pelvic organs, resulting in anomalous anatomical location and dysfunction (4). The etiology of POP is complex and diverse, and it is generally divided into microfactors, such as cyclin, matrix metal peptidases/tissue inhibitors of metalloproteinase (MMPs/TIMPs), and microRNAs (miRNAs); and macrofactors, such as age, vaginal delivery, obesity, and chronic respiratory diseases (4, 5). Despite extensive research on the etiology of POP, it has not been fully clarified. In addition, the clinical symptoms and effects of POP are diverse and tend to be generally polarized. Many women show asymptomatic POP, which may only be detected *via* a routine gynecological physical examination; such cases are not considered pathological and life and work are unaffected (5). However, symptomatic patients with POP may experience abnormal pelvic pressure, vaginal prolapse, dysfunctional urination and defecation, and sexual dysfunction (4, 5). Some patients have other complications, such as tissue ulcers, bleeding, and infection, which seriously affect quality of life and mental health (4, 5). It is reported that about 40% of women will suffer from pelvic organ prolapse (4), and this is predicted to climb as the population ages. By 2050, the number of women suffering from POP in the United States is projected to increase by approximately 50% (5). The total number of women undergoing POP surgery is anticipated to increase from 166,000 in 2010 to 245,970 in 2050 in United States (6). Besides, complications of POP cannot be ignored. At present, clinical treatment of POP can be classified as nonsurgical or surgical. Nonsurgical treatment may have complications such as tissue ischemia, necrosis or fistula formation, incarceration, bleeding, and infection (5), whereas complications from surgical treatment include mesh erosion, lower urinary tract symptoms, sexual dysfunction, and recurrent prolapse (5, 7). These phenomena cannot be ignored, and the therapeutic effect needs to be optimized. There remains a lack of accurate molecular biological intervention for POP. In this study, we reviewed and summarized the current knowledge of the molecular mechanisms of POP etiology associated with cyclins, MMPs/TIMPs, miRNAs, homeobox A11 (HOXA11), transforming growth factor β1 (TGF-β1), insulin-like growth factor 1 (IGF-1), fibulin 5 (FBLN5), lysyl oxidase-like 1 (LOXL1), oxidative stress, inflammatory response, estrogen, and biomarkers (Figure 4). This review aimed to clarify POP pathogenesis and provide more accurate potential biomarkers and intervention targets for the prevention, diagnosis, progression delay, and treatment of POP. However, as other, yet unknown, mechanisms may also lead to POP, further research is required in this field (Figures 1, 2).

## 2. Cyclin

Some cell cycle regulatory proteins are involved in the metabolism of collagen and other extracellular matrix (ECM) proteins, resulting in POP. For example, p53 is a tumor suppressor protein that monitors whether cells should continue their cell cycle, and p21, an inhibitor of G1 cyclin-dependent kinase, which regulates the initiation and progression of the cell cycle. By regulating p21, p53 inhibits the abnormal high-level proliferation and growth of cells in the ECM (8, 9). In one study, the expression of p53 and p21 decreased in prolapsed fibroblasts, and thus cells could not enter the inactive period, but they could enter the S phase from the late G1 stage (9). This led to decreased elastin synthesis and deposition, resulting in weakness or even loss of the supporting function of the pelvic connective tissue. Similarly, another study (10) on cell aging showed that the protein level of p53 was significantly lower in main ligament fibroblasts in patients with uterine prolapse compared with that of the control group,. The decrease in p53 protein expression in prolapsed fibroblasts may lead to higher proliferation activity and decreased synthesis and deposition of ECM components; the functional changes in supporting ligament fibroblasts were related to the mechanism of uterine prolapse. Further research (11) showed that p53 and p21 were lower in the uterosacral ligament (USL) of patients with POP than in non-POP patients, and that the levels of the two protein were positively correlated. This low expression is believed to lead to abnormal fibroblast proliferation in the pelvic support system, reduce the synthesis of elastin and other ECM components that should be secreted during the inactive period, and decrease connective tissue in support structures, such as the USL, which is considered to be linked to the appearance of POP. Furthermore, low expression of p53 in the USL increased the risk of uterine prolapse (POP-Q stage III-IV) 20.25 times, suggesting an effect on the metabolic balance of the ECM associated with the USL, resulting in insufficient elasticity of the ligament to support the pelvic organs (12). Therefore, through proper regulation of the expression of p53 and p21, the synthesis of ECM components such as elastin may be increased, and the macroscopic supporting structure of the pelvic floor may be strengthened, which would be beneficial for alleviating POP (Figure 3).

## 3. MMPs/TIMPs

MMPs are a family of zinc-dependent endopeptidases that participate in degrading a variety of ECM proteins, including collagen and elastin. TIMPs bind to MMPs in a 1:1 stoichiometric ratio, inhibiting MMP activity, and the MMP/TIMP ratio usually decides the degree of decomposition of ECM proteins and tissue reshaping (13). According to one study (14), type I collagen expression decreased and MMP-1 expression increased in the USL of patients with POP compared with that of the control group, suggesting an association with POP regulation. Another study (15) showed that MMP-2 and MMP-9 was more likely to be elevated in patients with POP with low amounts of collagen, indicating that an increase in these proteolytic enzymes is related to human POP disease. However, some different results were observed in other studies. For example, Gabriel et al. (16) discovered that when compared with the control, MMP-2 in the USL of patients with POP was enhanced, while MMP-1 was not. In contrast, another study (17) revealed that the expression of MMP-1 increased in patients with POP, but no difference in MMP-2 expression was found. Despite these differences in results, it is generally believed that an increase in proteolytic enzymes is closely related to prolapse. Further research (18) showed that women with POP had higher MMP-2 and lower TIMP-2 mRNA and protein expression in the USL than that of women without POP. Thus, an increase in MMP-2 expression and a decrease in TIMP-2 expression results in an increase in the degradation of ECM, which may lead to pelvic floor tissue defects and ultimately contribute to POP. A subsequent study (19) showed that in patients with POP, the expression level of MMP-2 gelatinase activity and ADAMTS-2 (procollagen I N-proteinase; a disintegrin and metalloproteinase with thrombospondin motifs) was increased in pelvic floor tissue. In addition, MMP-12 protein expression was upregulated in patients with POP in the proliferative phase of the menstrual cycle, while expression of TIMP-1–4 genes and TIMP-1 protein, which antagonizes MMPs, was decreased. These results suggest that an imbalance in the MMPs/TIMPs system may lead to connective tissue defects, which may result in POP. Therefore, it seems feasible that blocking ECM degradation by downregulating MMPs/TIMPs could be used to interfere in the occurrence and progression of POP (Figure 4).

## 4. miRNAs

miRNAs are noncoding small RNAs belonging to the family of gene regulatory factors that affect many biological functions, including cell proliferation, differentiation, apoptosis, organ development, and aging, by regulating the translation of mRNAs (20, 21). Recently, miRNAs have been the focus of considerable research, including in association with POP. For example, one study (22) confirmed that, compared to patients without POP, miR-19-3p expression increased, whereas expression of type I collagen and IGF-1 decreased in patients with POP. miR-19-3p contributed to vaginal fibroblast autophagy and apoptosis, and inhibited the production of type I collagen in POP *via* the protein kinase B (Akt) /mTOR/p70S6K pathway by targeting IGF-1. Another study (23) demonstrated that, compared with non-POP groups, the expression level of miR-4,429 in human USL fibroblasts from patients with POP decreased, and that miR-4,429 overexpression reduced the increase in phosphatase and tensin homolog (PTEN) expression and fibroblast apoptosis. In addition, compared with the control group (pelvic organ prolapse quantitation POP-Q < stage II), the expression of miR-30d and miR-181a in the USL of the POP group (POP-Q ≥ stage II) significantly increased (24), which shows that these miRNAs are related to POP. Both of these miRNAs are important post-transcriptional regulators of HOXA11, and abnormal expression of these miRNAs may also contribute to the pathogenesis of POP through pathways other than HOXA11 dysregulation. It can be seen that the decrease in miR-19-3p expression and the overexpression of miR-4,429 may lower the occurrence of POP by regulating the proliferation, differentiation, and apoptosis of fibroblasts. However, the decrease in miR-30d and miR-181a will negatively affect POP. These miRNAs may be used as biomarkers or potential molecular targets for clinical monitoring and intervention of POP.

## 5. HOXA11

HOXA11 is a transcription regulator that influences the development of urogenital embryos and the USL of mice and humans. POP is related to a decrease in HOXA11 and type III collagen expression, and an increase in MMP-2 expression in humans. In a mouse model with *HOXA11* deficiency, *in vitro* studies verified that The directional deletion of *Hoxa11* in mice led to an absent development of the USL. In addition, expression of HOXA11 decreased MMP-2 and increased type III collagen in the mouse fibroblasts, which benefitted collagen synthesis over degradation (25). A signal conduction defect in HOXA11 may lead to functional development or repair defects in the USL, which will alter the biomechanical strength of the USL and lead to the development of POP. Furthermore, the cytoarchitecture and smooth muscle content of the USL in women with prolapse were both reduced compared with that of women with normal pelvic support (26, 27). In addition, the number of cells in the USL of women with POP was reduced, as was the expression of HOXA11. HOXA11 can promote the proliferation of mouse fibroblasts and primary human USL cells *in vitro*, indicating that the decrease in HOXA11 in the USL of women with POP leads to a decrease in cell proliferation. Moreover, the expression of HOXA11 not only increased cell proliferation, but also decreased p53 expression, indicating that HOXA11 is involved in the regulation of the p53 inhibiting signal transduction pathway, promoting cell proliferation, and possibly reducing apoptosis (28). Using a Hoxa11-knockout (KO) model, the expression levels of type I and type III collagen and TIMP1 in the USL were shown to be significantly decreased. Meanwhile, the levels of pro-MMP-2, pro-MMP-9, and activated MMP-2 increased (29). These results indicate that Hoxa11-KO enhanced ECM degradation by regulating the expression level of the MMP/TIMP system, which is the likely mechanism by which the female pelvic floor support weakens. In addition, HOXA11 and TGF-β1 have a synergistic effect on the expression levels of collagen and MMPs (30), which jointly promote the synthesis of collagen, inhibit its degradation, and help inhibit POP. The decrease in ECM caused by the reduction of HOXA11 and TGF-β1 is a key factor in POP. Therefore, we speculate that the expression of HOXA11 can not only promote fibroblast proliferation by regulating the cell cycle, but also increase the ECM by regulating MMP/TIMP, which hinders the occurrence and progression of POP. Furthermore, the synergistic effect of HOXA11 and TGF-β1 also helps prevent the occurrence of POP. HOXA11 may be a potential biomarker for POP intervention.

## 6. TGF-β1

TGF-β1 is a profibrotic cytokine that is widely involved in fibrosis and degenerative fibrosis disease. The cytokine can induce fibroblast differentiation in cells from different tissues, leading to ECM deposition and secretion of paracrine and autocrine growth factors; moreover, TGF-β1 is important for fibroblast proliferation and ECM metabolism (31). However, in previous studies, the role of TGF-β1 in POP differed. Qi et al. (32) reported that TGF-β1 in pubic cervical fascia was negatively correlated with POP. In contrast, Mijerink et al. (33) reported that TGF-β1 in the vaginal wall was positively correlated with POP, while Leegant et al. (34) did not find any difference in TGF-β1 expression between USL samples (POP-Q ≥ II) and non-POP controls. However, in recent years, TGF-β1 has been shown to be negatively correlated with POP or its stages (30, 35–38). In one study (35), excessive mechanical stress and H2O2 treatment of USL fibroblasts reduced the level of TGF-β1, which was proven to reduce cell proliferation and ECM components, while increasing the ratio of MMP-2/TIMP2 mRNA. This indicated that the TGF-β1 signaling pathway could affect ECM remodeling by disrupting the MMP/TIMP balance. Another study (36) showed that TGF-β1 pretreatment could stimulate TIMP-2 synthesis and inhibit MMP-2/9 activity through the TGF-β1/Smad3 signaling pathway, reducing the loss of ECM in POP USL fibroblasts subjected to excessive mechanical stress. A recent study (37) showed that the expression of TGF-β1 was similar between symptomatic patients with POP and the controls, who did not show any signs of prolapse. Compared with moderate/mild cases, TGF-β1 was more commonly expressed in severe prolapse, suggesting its association with the progression of POP (i.e., repair after injury). Another recent study (38) showed that the expression of phospho-p44/42 and TGF-β1 declined in patients with POP than without POP and it was positively related to collagen expression; the low-level of expression was deemed to be linked to the presence of POP. In addition, further study (39) proposed that the crosstalk between calpain and TGF-β1 activated the TGF-β1 Smad2/3 and non-Smad (Akt) pathways, enhancing type I collagen synthesis in human lung fibroblasts and pulmonary fibrosis. This may also be one of the mechanisms of POP. According to the above research findings, TGF-β1 may have a role in the occurrence and/or progression of POP by negatively regulating collagen synthesis and interfering with ECM metabolism. However, based on some differences in the findings of the above studies, further research is needed to clarify its molecular biological mechanism.

## 7. IGF-1

IGF-1 belongs to the insulin-like growth factor family, which includes insulin-like polypeptides mainly synthesized by the liver. IGF-1 can regulate ECM metabolism and various biological processes, such as cell proliferation, differentiation, and apoptosis (40, 41). One study (40) have shown that IGF-1 levels in vaginal wall tissues were lower in patients with POP than in non-POP controls, and it induced the proliferation of vaginal wall fibroblasts, activated mitogen-activated protein kinase (MAPK) and nuclear factor-κ-gene binding (NF-κB) pathways, promoted the metabolism of type I and III collagen another study (42) reported that IGF-1 can be used as an inhibitor of apoptosis and it may also stimulate fibroblasts to release ECM molecules, such as polysaccharides and proteins. Further study (22) proposed that, the expression of IGF-1 decreased in the vaginal wall of patients with POP, and it inhibited autophagy and apoptosis and promoted expression of type I collagen, affecting the metabolism of the ECM by activating the Akt/mTOR/p70S6K pathway in vaginal fibroblasts. In all, IGF-1 may stimulate the proliferation of fibroblasts; activate Akt/mTOR/p70S6K, MAPK, and NF-κB pathways; promote collagen synthesis; and block the occurrence and progression of POP.

## 8. FBLN5 and LOXL1

FBLN5 is a calcium-dependent elastic fiber-related protein belonging to the short fibrin family (43). LOXL1 belongs to the family of lysyl oxidases, and it activates tropoelastin through specific localization and binding with the fibrin-5 domain. Tropoelastin is converted to mature elastin through covalent crosslinking, which is crucial for the synthesis and assembly of elastic fibers (44). However, as discussed here, studies on FBLN5 and LOXL1 in humans have produced conflicting results. Kluetke et al. (45) measured the elastin protein content in the USL using western blot analysis and LOXL1 and FBLN5 mRNA levels using real-time quantitative polymerase chain reaction (RT-qPCR). They found that compared with women with normal pelvic support, the level of LOXL1 in the USL biopsy of the POP group (POP-Q ≥ III) was reduced, while that of FBLN5 was increased, and the elastin content decreased significantly. In a similar study using the same techniques, Jung et al. (46) found that the mRNA and protein expression levels of FBLN5 significantly decreased in the advanced POP (POP-Q III-IV) group, while that of LOXL1 increased compared with that of the non-prolapsed group. Although both authors used similar tissue samples and detection techniques, they observed opposite results. Kluetke et al. (45) surmised that the increased expression of FBLN5 might be a secondary effect of tissue injury in patients with POP; it has been shown that the level of fibrin-5 mRNA increases when lung tissue is injured by elastase (47). Jung et al. (46) interpreted the increase in LOXL1 expression as a compensatory mechanism secondary to abnormal crosslinking, although the structural disorder of elastin has not been studied. Recently, Garcia et al. (48) used western blotting and an enzyme-linked immunosorbent assay (ELISA) to quantify LOXL1 and FBLN5 protein expression in vaginal secretions of women with and without POP; LOXL1 protein expression was higher in patients with POP, while the expression of FBLN5 did not significantly differ between the two groups. The increase in LOXL1 expression was believed to reflect a compensatory mechanism in women with POP. This also seems to be in agreement with the findings in most studies that FBLN5 is reduced in POP, although the statistical significance has not been demonstrated. In another study (49), immunohistochemical staining revealed a decrease in expression of FBLN5 and LOXL1 in abdominal hysterectomy ligament samples of the POP group (POP-Q ≥ II) compared with the control group, suggesting that this low expression may be important in weakening the supporting structure of the pelvic floor. Similarly, Takacs et al. (50) showed that FBLN5 mRNA and protein levels were significantly lower in women with anterior vaginal wall prolapse than in women without anterior vaginal wall prolapse, and this low expression was considered to be involved in POP. Alarab et al. (51) reported that LOXL1 mRNA and protein expression in the vaginal tissue of POP group was lower compared with that in asymptomatic control group, which may have led to assembly defects in the pelvic tissue. In addition, a large number of animal model experiments have also been carried out to help elucidate the possible role and related mechanism of FBLN5 and LOXL1 in POP, as discussed below.

### 8.1. FBLN5

A previous study (52) showed that compared with wild-type (WT) mice, *Fbln5*-deficient mice exhibited the same phenotype as that of women with POP, such as descending and extending vagina and cervix, bulging vaginal wall, increased genital hiatus, and bulging bladder in some cases. As young as 3 months old, virgin *Fbln5*-KO mice developed a bulging urogenital system. By 6 months, 92% (33/36 cases) of the female *Fbln5*-KO mice had POP. Severe prolapse occurred in mice aged ≥6 months. In this case, a defect in *Fbln5* was thought to be related to POP. In addition, in *Fbln5*-KO mice, pelvic floor suspension connective tissues, such as the USL, were either missing or stunted. Moreover, in the older (6 months old) *Fbln5*-KO mice, the USL in females was either missing or weakened, indicating that FBLN5 is related to defective congenital development, weakness, and acquired repair of the pelvic floor support structure. Another study (53) showed that compared with WT mice, approximately 90% of *Fbln5*-KO mice prolapsed with age. Compared with non-pregnant mice, *Fbln5*-KO prolapsed mice showed biomechanical changes, such as decreased hardness, decreased maximum load, and increased expansibility, indicating that impaired elastin function and pelvic floor biomechanical changes led to prolapse. In addition, compared with those in WT mice, MMP-9 and MMP-2 levels were enhanced in the vaginal tissues of mature *Fbln5*-KO mice, the pelvic organ support deteriorated progressively, and 90% of *Fbln5*-KO mice prolapsed at the age of 6 months (54). Notably, the lack of *Fbln5* expression in the vaginal wall not only contributed to genetic defects in the synthesis and assembly of elastic fibers, but it also led to increased protease activity and elastin decomposition, which inhibited the repair or synthesis of new elastic fibers. This was also believed to be the cause of the failure of matrix regeneration in connective tissues supporting the pelvic floor and the occurrence of POP. By negatively regulating the interaction of β1 integrin and fibronectin in the vagina of mice, FBLN5 inhibited the pro-MMP-9 and active MMP-9, increased the density of elastic fibers, and improved collagen fibers, which was not conducive to POP (15). In addition to the upregulation of MMP-2 and MMP-9, serine protease inhibitors (serpina1a [a1-antitrypsin] and elafin) were reduced in vaginal tissues of POP and were dysregulated in the epithelium of *Fbln5*-deficient mice. MMP-9 and a trypsin-like serine protease were upregulated in the *Fbln5*-KO mice. PRSS3, a major extra-pancreatic trypsinogen, is expressed in human vaginal tissues (55). It is suggested that other proteases and protease inhibitors are also involved in POP, and the balance between proteases and their inhibitors may provide insight into POP in humans and mice. The histological changes were further verified, for example, in *Fbln5*-deficient mice (55), no change was seen in the size of perineal eminence over the course of the gravidity. However, elastic fiber breakage and inflammatory infiltration appeared in the vaginal wall at the beginning of the postpartum period (2–24 h). The seriousness of POP increased nearly 1 week after delivery, which further showed that the deletion of *Fbln5* contributed to failed repair of the birth-related injury. A study of vaginal dilation using a balloon to simulate labor (56) showed that the activities of MMP-2 and MMP-9 increased in the vaginal wall of non-gravid and gravid animals, with noticeable fragmented and broken elastic fibers in the vaginal wall. Compared with WT mice, vaginal dilation led to accelerated POP in non-pregnant *Fbln5*-KO mice, which never recovered. Similar to the results of previous studies, it can be seen that FBLN5 is also important for the protection and recovery of labor- and elastase-induced prolapse. In addition, another study of vaginal dilatation-simulated childbirth (57) showed that the levels of markers(p53 and γ-H2Ax) of cell senescence decreased in WT mice 1 week after distention, but not in *Fbln5*-KO mice. This suggests that WT mice can carry out cell proliferation and repair 1 week after injury; however, in *Fbln5*-KO mice, aging markers cannot be downregulated to repair tissues, leading to the accumulation of cell senescence and destruction of the ECM and connective tissue, which may be a potential injury mechanism of POP. Furthermore, PTK7 and β-catenin, which are involved in elastin production after vaginal mechanical expansion, were upregulated in WT mice, but not detected in *Fbln5*-KO mice, indicating that these proteins may also participate in POP in *Fbln5*-KO mice (58). In summary, FBLN5 deficiency seems to affect the pathology of POP by not only promoting an increase in markers of cell senescence and proteases/protease inhibitors, but also by reducing β1 integrin and fibronectin.

### 8.2. LOXL1

Liu et al. (59) showed that spontaneous pelvic floor disorder developed slowly in nulliparous Loxl1-KO mice. At the age of 1 year, approximately 50% of cases showed signs of pelvic organ decline, whereas WT animals did not show signs of POP or decline at 18 months. LOXL1 is associated with POP. Besides, POP occurs in mice with a *Loxl1* mutation after giving birth to either the first or second cub; however, no prolapse occurres in WT mice in the same age range (3–7 months). Parturition may be the most important trigger of POP in female *Loxl1*-KO mice. Lee et al. (60) also confirmed the above conclusion and reported that *Loxl1*-deficient mice could not rebuild standard elastic fibers during the reshaping of reproductive tract connective tissues after pregnancy and delivery, which contributed to POP. Liu et al. (61) showed that *Loxl1*-deficiency prevented deposition of normal elastic fibers in the uterus after delivery and demonstrated pathological manifestations of elastic fiber functional defects, such as POP, skin relaxation, and vascular abnormalities. Thus, LOXL1 plays an important part in the synthesis and assembly of elastic fibers in the process of pelvic floor injury repair. Alperin et al. (62) verified the change in mechanical properties in the vagina; compared with age-matched WT animals, *Loxl1*-deficient animals exhibited poor biomechanical properties of the vaginal supporting tissue complex. This characteristic was thought to be due to overall structural defects in the connective tissue rather than the loss of vaginal support itself. Morphometric analysis of elastic fibers in the cultivation of vaginal tissues demonstrated that compared with WT mice, the aspect ratio of elastic fibers in *Loxl1*-KO mice at 3 weeks of age was significantly smaller, proving that there was an increased number of shorter and broken elastic fibers (63). This showed that there was continuous elastic decomposition activity in the culture tissue of *Loxl1*-KO cells, suggesting that both quantitative and qualitative facets of elastic fibers may be involved in the pathophysiology of POP. The results of a separate study (37) showed that compared with the levels in WT mice, the total and unit cell amounts of elastin and unit cell amount produced by non-epithelial vaginal cells in LOXL1-deficient mice were significantly reduced, while the ratio of MMP-9/TIMP-1 was relatively high. A recent study by Couri et al. (64) indicated that in contrast to WT mice, the mRNA levels of chemokine C-X-C motif ligand 12 and chemokine C-C motif ligand 7 (mediators of inflammatory response) were differentially upregulated in the tissues of virgin Loxl1-KO mice, and they were significantly upregulated in the vagina, urethra, bladder, and rectum of pregnant Loxl1-KO mice. However, in Loxl1-KO mice after vaginal childbirth, cytokines were expressed differently in terms of time, tissue, and concentration. Furthermore, the urethra and vagina may be especially susceptible to delivery injury. Based on the above studies, we concluded that LOXL1 deficiency may play a role in elastin synthesis and assembly by downregulating TGF-β1 activity and upregulating MMPs/TIMPs, chemokine C-X-C motif ligand 12, and chemokine C-C motif ligand 7 in POP. Moreover, LOXL1 deficiency seems to be particularly important in the repair process after an injury, such as a childbirth-related injury.

In summary, we can see that the results using animal models are consistent, suggesting that defects in Fbln5 and Loxl1 are involved in the pathological process of POP through different mechanisms. However, the different results reported for studies in humans may be related to the complexity of the structure and mechanisms at play in the human body, but differences in research design cannot be ruled out; thus, further research is needed.

## 9. Oxidative stress

Oxidative stress is caused by an imbalance in the oxidative and antioxidant defense system in cells, tissues, or organs, which leads to the accumulation of reactive oxygen species (ROSs), and oxidative damage of DNA, lipids, and proteins (65). The levels of oxidative stress biomarkers are higher in the pelvic floor of patients with POP than in control patients; these include isoprostanes (66), 8-hydroxy-2-deoxyguanosine (8-OHdG), 4-hydroxy-2-nonenal (4-HNE) (67), advanced glycation end-products (AGEs) (68–70), and mitofusin2 (Mfn2) (71–75). In contrast, the levels of antioxidant markers are lower in patients with POP than in control patients; these include glutathione peroxidase, superoxide dismutase (76), and nuclear factor erythroid-2-related factor 2 (Nrf2) (77). This may lead to damage of the pelvic floor tissue and contribute to the development of POP by regulating the MMP/TIMP balance (66) and inducing mitochondrial apoptosis (67) and other signal transduction pathways (68–79). One study (66) showed that the level of isoprostanes was higher in the fibroblasts of the main ligament and urine samples of women with uterine prolapse than in women without uterine prolapse, and MMP-2 mRNA expression in the main ligament of patients with uterine prolapse significantly increased. Oxidative stress is involved in POP, especially uterine prolapse, through direct regulation of the ECM or post-transcriptional regulation of MMP-2 by isoprostanes. Oxidative stress markers 8-OHdG and 4-HNE were markedly higher in the USLs of patients with POP (POP-Q III or IV stage) than in controls (67). In addition, a significant positive correlation was observed between oxidative stress markers and mitochondrial apoptosis markers in pelvic supporting connective tissue of patients with POP (67), indicating that oxidative stress weakens the pelvic-supporting tissue in patients with POP and suggesting a possible mechanism for mitochondrial apoptosis in the USL. Chen et al. (68) reported that the level of AGEs was higher in prolapsed tissues, while that of type I collagen was lower. Further experiments (69) showed that MMP-1 levels were higher in human vaginal fibroblasts of patients with POP than in controls. In addition, AGEs inhibited vaginal fibroblast proliferation in patients with POP and decreased the expression of type I collagen *via* receptor of advanced glycation end products (RAGE) and/or MAPK and nuclear factor-κB (NF-κB) pathways, which affects ECM metabolism and weakens the supporting structure of the pelvis. Vetusch et al. (70) showed that compared with the non-POP group, the anterior vaginal wall in the POP group exhibited a disordered normal myometrium structure and had upregulated AGE, extracellular signal-regulated kinase 1/2 (ERK1/2), Smad-2/3, MMP-3, and type III collagen in the myometrium. AGEs, ERK1/2, and Smads 2/3 may participate in the pathogenesis of POP. Mfn2 is a transcription product of oxidative stress and a crucial regulator of mitochondrial fusion and division, which is related to proliferation, apoptosis, and signal transduction (71, 72). The level of Mfn2 increased in USL fibroblasts obtained from patients with POP and decreased procollagen, and the increase in Mfn2 inhibited fibroblast proliferation and the cell cycle by regulating the Ras/Raf/ERK pathway (73–75). A decline in antioxidant defense ability is also involved in POP. Compared with the control group (POP-Q ≤ II stage), the expression of OHdG and 4-HNE in the main ligament in the POP group (POP-Q III-IV) were higher. However, the protein levels and enzyme activities of mitochondrial superoxide dismutase (MnSOD) and glutathione peroxidase 1 (GPx1) were lower. Compared with mild POP, the oxidative damage to pelvic-supporting ligaments in female patients with severe POP increased, while the antioxidant defense ability decreased. Thus, the accumulation of ROSs and a decrease in antioxidants may be involved in development of POP. In addition, Nrf2 and GPx are key transcription factors implicated in controlling the anti-oxidant defensive system (76). Lin et al. (77) analyzed discussed the expression of cyclooxygenase-2 (COX-2) and Nrf2/GPx3 in the lamina propria of the anterior vaginal tissue of women with or without POP. They showed that the arrangement of collagen fibers in the anterior vaginal wall was disordered and discontinuous in the POP group relative to the non-POP patients. The levels of Nrf2, GPx3, TIMP1, and type I and III collagen decreased significantly, while those of COX-2, prostaglandin E2, and MMP2 increased significantly in the POP group compared with those of the control group (77). These results demonstrated that oxidative stress and inflammation are closely related to POP. When exogenous H_2_O_2_ was used to treat primary cultured sacral ligament fibroblasts to establish the original oxidative stress cell model (78), it was concluded that oxidative stress might participate in the disorder of collagen metabolism by inhibiting the synthesis and metabolism of collagen or indirectly regulating MMPs, TIMPs, and TGF-β1. This had a negative effect on ECM production, destroying the pelvic floor support network, and likely participating in the pathophysiology of POP. Activation of the phosphatidylinositol-4,5-bisphosphate 3-kinase (PI3K)/Akt signaling pathway leads to the accumulation of ROSs, promotes the aging and apoptosis of fibroblasts in pelvic tissues and a decrease in type I collagen, and leads to the relaxation and dysfunction of pelvic support (79). In addition, AGEs regulate the miR-4,429/PTEN/PI3K/Akt pathway, inducing apoptosis of human USL fibroblasts, which contributes to POP (23). Taken together, these studies indicate that oxidative stress leads to fibroblast apoptosis through complex molecular biological mechanisms and interferes with the metabolism of ECM, leading to the dysfunction of the pelvic floor support network. By monitoring oxidation and antioxidant markers in the pelvic floor support system, effective intervention programs may be formulated to block signaling pathways and ultimately prevent the pathophysiology of POP.

## 10. Microenvironment of the inflammatory response

In an examination of the inflammatory environment in the pelvic floor, patients with POP exhibited a higher level of inflammation in vaginal tissues than did controls with non-prolapsed tissues, which confirmed that the extensive changes in the inflammatory environment in the pelvic floor are part of the pathogenesis of POP (80). A further study (79) showed that COX-2, prostaglandin E2, and MMP-2 are more highly expressed in POP patients than in non-POP patients. The release and expression of inflammatory cytokines in the front vaginal wall in the POP group were greater than in the control group, which may affect collagen metabolization and lead to POP. In addition, the interaction of inflammation and oxidative stress further worsened the pelvic floor branch system. In an analysis (81) of severe anterior vaginal wall prolapse (AVP) tissue at the single-cell level, members of the FOS/JUN family, hyaluronan (HA) degradation genes, HA receptors, and collagen endocytic receptors (MRC1 and MRC2), which are thought to be related to the inflammatory response, were all upregulated in macrophages in POP samples. Moreover, IL18-CD48 pro-inflammatory cytokine interactions were discovered in fibroblasts and immune cells, and IL1B–IL1R1 inflammatory activators and their interactions were discovered in smooth muscle cells. These findings further support that inflammation is involved in POP and that the inflammatory microenvironment could be a key factor in POP intervention; however, further research is needed to enrich our understanding of the molecular biological mechanisms involved.

## 11. Estrogen

The role of estrogen in POP is controversial. It is generally believed that the high incidence of POP after menopause is due to the rapid decline in female estrogen secretion, the weak supporting structure of the pelvic floor, the downward displacement of pelvic organs, and the emergence of pelvic floor dysfunction (82, 83). However, a randomized, double-blind, placebo-controlled, multicenter study by Marschalek et al. (84) showed that there was no difference in subjective prolapse-related complaints over a 6 week period in patients using preoperative vaginal estrogen cream and placebo cream groups. This suggested that preoperative locally applied estrogen does not ameliorate prolapse-associated symptoms in postmenopausal women with symptomatic POP. However, the findings also indicated that longer observation time intervals may be needed. Jackson et al. (85) conducted a double-blind, placebo-controlled study for 6 months on postmenopausal women with stress urinary incontinence treated with estradiol valerate. The study showed that compared with the placebo control group, total collagen, mature crosslink histidinohydroxy lysino norleucine, AGEs, and non-fluorescent compound-1 (NFC-1) decreased in the periurethral biopsy tissues of the treated group. However, levels of pro-MMP-2 and immature crosslinked hydroxylysinoketo-norleucine increased significantly, while collagen type I/III ratios did not differ significantly between groups. These results suggested that estrogen therapy leads to increased protease activity, degradation of collagen and AGEs, and increased levels of immature protein. Furthermore, the results suggested that aged collagen degradation was only an initial reaction to estrogen stimulation.; a prolonged exposure interval may be needed to demonstrate the whole collagen content In an *in vitro* study (86), 17β-estradiol inhibited the proliferation of fibroblasts from the main ligament of patients with and without POP, but it was more evident in patients with POP. A decrease in fibroblast renewal may reduce the production of collagen, elastic fibers, and other ECM proteins, and weaken the supporting force of the main ligament, thereby contributing to POP. Erika et al. (87) reported that ongoing hormone treatment is highly associated with the descent of the rectal ampulla as well as Gh + Pb (genital hiatus + perineal body), as detected by ultrasound. Hormone therapy may increase rather than decrease the descent in pelvic organs. In contrast, Clark et al. (88) showed that after 5 months of estradiol treatment, collagen type I and III in pelvic floor connective tissue increased, and cystatin C, a proteinase inhibitor that prevents the degradation of collagen, also increased, suggesting that estrogen decreases the degradation of collagen by increasing cystatin C. Nunes et al. (89) showed that in a 30-day double-blind trial of estrogen and placebo in post-menopausal women, both with and without POP, the levels of hyaluronic acid and chondroitin sulfate at the top of the vagina were higher in women treated with estrogen than in those treated with placebo. Both hyaluronic acid and chondroitin sulfate are glycosaminoglycans, which are important components of the ECM. These results showed that estrogen increased the production of ECM. Other studies have shown that hyaluronic acid induces the vitality of fibroblasts and the production of collagen in the ECM (90, 91). In addition, estrogen applied topically for 6 weeks in postmenopausal women with POP, increased the thickness of epithelial and muscular layers of the vaginal wall at the macro-level and enhanced the synthesis of mature type I collagen at the micro-level (92). In contrast, type III collagen did not change significantly, resulting in an increase in the ratio of type I/III collagen and a decrease in the activity of the collagen-degrading enzyme MMP-9. Furthermore, 17β-estradiol inhibited expression of Mfn2 at the mRNA and protein level and increased fibroblast proliferation and procollagen 1A1/1A2/3A1 synthesis, while also increasing the expression of estrogen receptor and G protein-coupled receptor 30 in USL fibroblasts (93). A recent study (94) has shown that 17β-estradiol increased the protein and mRNA levels of anti-apoptosis poly-ADP-ribose polymerase (PARP1) and B-cell lymphoma-2 (Bcl-2). At the same time, the expression of estrogen receptor alpha, a target of poly-ADP-ribosylation of PARP1, was enhanced, and apoptosis and death of USL fibroblasts subjected to mechanical stress *in vitro* were reduced. In summary, there are significant differences in the efficacy of estrogen in pelvic floor support tissue, but further studies are needed to better understand the role of estrogen in the pathophysiology of POP.

## 12. Other potential biomarkers

Other potential biomarkers have been shown to be associated with POP. For example, Deng et al. (95) applied a non-targeted metabolomics approach using ultra-high performance liquid chromatography coupled with quadrupole-time-of-flight mass spectrometry (UHPLC-Q-TOF-MS) to analyze and compare serum and urine of patients with POP with that of controls. And discovered that glycerophosphocholine, L-pyroglutamic acid, and inosine were increased while citrate was decreased in both serum and urine samples of patients with POP, which may be related to collagen synthesis or degradation, further lead to POP development This suggests that these six metabolites may be used as discriminatory POP biomarkers. Shama et al. (96) used capillary electrophoresis-tandem mass spectrometry (CE-MS) to study 17 amino acids in the pelvic connective tissue of patients with POP, and found that methionine, histidine, and glutamine levels were significantly higher in the POP group when comparing to their levels in none-POP group, and their increased levels could relate to the development of POP. A proteomic study by Sun et al. (97) using two-dimensional electrophoresis and matrix-assisted laser desorption/ionization TOF-MS (MALDI-TOF-MS) analysis of USL proteins of patients with POP and controls identified eight proteins (flavoprotein, apolipoprotein A-I, actin, transgelin, cofilin-1, cyclophilin A, myosin, and galectin-1) that were downregulated in the POP group. RT-qPCR was used to validate this conclusion at the mRNA level. These proteins may be involved in the pathophysiology of POP. Further proteomic analysis of the etiology and pathogenesis of POP using HPLC-MS/MS, iTRAQ, and ingenuity pathway analysis (IPA) techniques described by Li et al. (98), revealed five differentially expressed proteins (fibromodulin, collagen alpha-1 [XIV] chain, calponin-1, tenascin, and galectin-1) that appear to be involved in Metabolic mechanisms of the pelvic floor connective tissue. Wang et al. (99). further studied plasma samples using protein array analysis and ELISA in patients with POP and controls and found that the mean plasma levels of heat shock protein 10, zinc finger CCCH domain-containing protein 8, and unc-45 myosin chaperone A were lower than those in healthy controls, these proteins are diagnostic biomarkers for POP. In addition, a number of gene alterations affecting the genetic predisposition of POP have been studied. Certain candidate genes (*COL3A1, COL18A1, LAMC 1, MMP 1, MMP 3, MMP 9, MMP10, ZFAT*) (100–108) may be mediators of POP occurrence; the *COL1A1* rs1800012 polymorphism did not show a significant correlation with POP (109, 110). Contrary to the view outlined above, Cartwright et al. (111) reviewed some genetic correlation studies prior to May 1, 2014, and concluded that the rs1800012 polymorphism in the *COL1A1* gene was linked to anatomic prolapse. However, candidate genes *COL3A1, LAMC 1, MMP 1, MMP 3, and MMP 9* failed to show a significant predisposition to POP. A recent meta-analysis (112) of data related to the genetics of POP, collected between January 1, 2015 and November 1, 2020, yielded the same conclusions as those of Cartwright et al. (108). Furthermore, meta-analyses of the candidate genes *COL18A1* (collagen type 18), *ZFAT*, and *MMP10* did not yield significant predisposition to POP. Notably, some previous studies (113–116) found that *ESR* (estrogen receptor)*, PGR* (progesterone receptor), and *Fbln5* are also involved in the pathophysiological mechanism of POP. Similarly, a meta-analysis report of Allen-Brady et al. (112) concluded that there is a significant correlation between *ESR1* RS2228480, *FBLN5* RS12589592, and *PGR* RS484389 and POP. Several other genetic biomarkers have also been explored; for example, Xie et al. (117) carried out an RNA-Seq study of USL specimens from patients with POP and controls and identified 81 POP signature genes. In addition, some ECM-related candidate genes, such as *COMP, NDP, and SNAI2*, were suggested to contribute to the pathological process of POP. Furthermore, components in neuroactive ligand-receptor interactions and the Wnt receptor signaling pathway were also indicated to be involved in the pathogenesis of POP. A single-cell transcriptome study (81) of severe AVP (POP-Q ≥ stage III) found abnormal gene expression in different cell types, including genes encoding ECM molecules (*FN1, LUM*, and *DCN*) or receptors for cellular uptake of HA and collagen (*LYVE1* and *MRC2*), which were widely upregulated in most cell types in the POP samples. In addition, two types of collagen endocytic receptors (*MRC1* and *MRC2*), HA degradation genes (*HYAL2, HYAL3*), and HA receptor (*LYVE1*), which are believed to regulate inflammation by converting signals from the ECM, were upregulated in macrophages in POP samples. Thus, fibroblasts and macrophages may play a significant role in the dysregulation of the ECM and immune disorders associated with POP. In a genome-wide association study using data from Iceland and the United Kingdom Biobank, Olafsdottir et al. (118) reported the discovery of eight sequence variants at seven loci associated with POP. These included *rs3820282–T* located in intron1 of *WNT4*, *rs12325192* located near *SALL1*, *rs9306894* located in the *3′-UTR of GDF7*, *rs1247943* close to *TBX5*, r*s7682992–T* close to the *FAT4* gene, r*s72624976* located in the *3′-UTR of IMPDH1*, and *rs3791675* and *rs1430191* partly located in and near *EFEMP1*. In addition, Natalia et al. (119) performed a genome-wide association meta-analysis and identified 26 loci significantly associated with POP, of which 7 loci are as described in the above studies, and the others are previously unidentified potential candidate genes, such as *VCL, CHRDL2, LOXL1-AS1, DUSP16, CRISPLD2, ADAMTS5, KLF13, MAFF in 2p24.1, 10q22.1, 11q13.4, 12p13.2, 16q24.1, 15q24.1, 15q13, 21q21.3*, and *22q13*, as well as *ACADVL, PLA2G6, and HOXD13*. In summary, a large number of potential POP biomarkers have been identified using metabolomics, proteomics, and genetic susceptibility, although some remain controversial. The described studies contribute to our global understanding and provide new insights into the molecular mechanisms of POP, further opening new avenues for future research.

## 13. Conclusion

POP is a concerning gynecological disease that occurs in middle-aged and senior women, and its molecular mechanism is complex. By exploring the mechanisms of various molecules associated with POP, we were able to summarize a large number of potential key molecular targets and/or signaling pathways involved in the evolution of POP. There is a current lack of accurate clinical molecular biological interventions to prevent, diagnose, progression delay, and improve the treatment of POP. We suggest that key molecular targets can be used to develop simple, rapid, and effective detection techniques for early screening in community health care centers to identify high-risk individuals. In addition, we propose reasonable preventive measures associated with risk factors to reduce the incidence of POP, as well as identify asymptomatic POP patients. Finally, we provide scientific evidence for early diagnosis, intervention, and treatment of POP to delay progression of the disease and improve quality of life. The key molecular targets and signaling pathways associated with POP can be used to develop new biological meshes related to pelvic floor surgery, reduce complications such as postoperative recurrence, and develop molecular targeted drugs to accurately strengthen the pelvic floor structure, or even reverse prolapse. Finally, further research studies are needed, especially to address existing contradictions in the literature regarding the potential molecular mechanisms of POP.

## Author contributions

XL conceptualized article. XW wrote first draft. TL plotted the figures. All authors contributed to the manuscript revision and approved the submitted version. All authors agreed to be accountable for all aspects of the final manuscript.

## Funding

This work was supported by the National Natural Science Foundation of China (grant number 81971365), Key Research and development program of Shanxi province (international scientific and technological cooperation; grant number 201903D421060), Research Project Supported by Shanxi Scholarship Council of China (grant number HGKY2019095), Fund Program for the Scientific Activities of Selected Returned Overseas Professionals in Shanxi Province (grant number 20200007).

## Conflict of interest

The authors declare that the research was conducted in the absence of any commercial or financial relationships that could be construed as a potential conflict of interest.

## Publisher’s note

All claims expressed in this article are solely those of the authors and do not necessarily represent those of their affiliated organizations, or those of the publisher, the editors and the reviewers. Any product that may be evaluated in this article, or claim that may be made by its manufacturer, is not guaranteed or endorsed by the publisher.
